# Supplementary material for: Coloration Modeling and Processing of Commodity Plastic Buttons in Supercritical Carbon Dioxide
Source: Materials (Basel). 2023 Jan 17;16(3):907. doi: 10.3390/ma16030907 (PMC9917541; doi:10.3390/ma16030907)
Supplement: Supplementary file 1 [file materials-16-00907-s001.zip › materials-2127429-supplementary.pdf]

materials

Supplementary Data

Coloration Modeling and Processing of Commodity Plastic Buttons in Supercritical Carbon Dioxide

Table S1. The experimental *K/S* values for acryl button at pressure, temperature, and treatment time, along with CO_2 density and solubility of dye at every pressure and temperature.

K/S	*P*/MPa	*T*/°C	*t*/min	ρ/mol・m^{-3}	*y*
27.47	8	40	30	6314.4	1.18E-07
36.51	8	40	40	6314.4	1.18E-07
23.72	8	40	60	6314.4	1.18E-07
18.83	8	50	30	4980.3	7.25E-08
32.65	10	40	30	14283	6.90E-06
33.41	10	40	40	14283	6.90E-06
39.88	10	40	50	14283	6.90E-06
27.63	10	40	60	14283	6.90E-06
34.81	10	45	30	11321	2.90E-06
25.61	10	45	40	11321	2.90E-06
22.74	10	45	60	11321	2.90E-06
26.89	10	50	30	8732.8	1.10E-06
24.96	10	50	60	8732.8	1.10E-06
20.14	10	55	30	7386.4	6.61E-07
27.42	10	55	40	7386.4	6.61E-07
22.48	10	55	60	7386.4	6.61E-07
32.87	12	40	30	16309	1.34E-05
29.07	12	40	40	16309	1.34E-05
25.75	12	50	30	13286	8.34E-06
29.56	14	40	30	17343	1.82E-05
26.18	14	45	30	16371	1.77E-05
46.03	14	50	40	15273	1.64E-05

Table S2. The experimental *K/S* values for polyester button at pressure, temperature, and treatment time, along with CO_2 density and solubility of dye at every pressure and temperature.

K/S	*P*/MPa	*T*/°C	*t*/min	ρ/mol・m^{-3}	*y*
27.47	10	115	30	3906.8	1.03E-06
28.36	10	115	60	3906.8	1.03E-06
29.31	10	120	20	3801.6	1.18E-06
25.89	10	120	30	3801.6	1.18E-06
30.45	10	120	40	3801.6	1.18E-06
27.58	10	120	60	3801.6	1.18E-06
30.19	10	125	60	3704.4	1.35E-06
30.32	12	115	40	4931.6	2.66E-06
33.1	12	120	30	4778.6	2.96E-06
39.44	12	120	60	4778.6	2.96E-06
26.63	12	125	20	4639.2	3.30E-06
40.56	12	125	30	4639.2	3.30E-06
41.74	14	110	30	6274.1	5.65E-06
34.89	14	110	40	6274.1	5.65E-06
39.44	14	110	60	6274.1	5.65E-06
42.48	14	115	30	6037.6	6.06E-06
31.2	14	115	60	6037.6	6.06E-06
33.18	14	120	20	5826.2	6.55E-06

31.34	14	120	30	5826.2	6.55E-06
35.77	14	120	40	5826.2	6.55E-06
42.48	14	120	60	5826.2	6.55E-06
42.11	14	125	20	5635.5	7.13E-06
36.41	14	125	30	5635.5	7.13E-06
42.99	14	125	40	5635.5	7.13E-06
45.3	14	125	60	5635.5	7.13E-06
34.22	16	110	40	7509.1	1.18E-05
36.6	16	120	30	6923	1.31E-05
44.32	16	120	40	6923	1.31E-05
35.24	16	120	60	6923	1.31E-05

Table S3. The experimental K/S values for casein button at pressure, temperature, and treatment time, along with CO_2 density and solubility of dye at every pressure and temperature.

K/S	***P*/MPa**	***T*/°C**	***t*/min**	**ρ/mol・m^{-3}**	***y***
31.13	8	80	30	3643.2	1.15E-07
28.42	8	80	60	3643.2	1.15E-07
28.83	8	100	60	3210.1	2.10E-07
28.89	8	110	40	3045	2.90E-07
33.38	10	80	20	5035.3	4.83E-07
34.55	10	80	30	5035.3	4.83E-07
28.83	10	80	40	5035.3	4.83E-07
32.37	10	80	60	5035.3	4.83E-07
32.19	10	90	30	4611.1	5.71E-07
32.53	10	90	40	4611.1	5.71E-07
27.15	10	100	30	4284.6	7.10E-07
33.89	10	100	60	4284.6	7.10E-07
38.17	10	110	60	4021.2	9.08E-07
35.37	12	80	40	6742.5	1.77E-06
31.62	12	80	60	6742.5	1.77E-06
30.19	12	90	60	6020.2	1.81E-06
30.99	12	100	20	5500.4	2.03E-06
34.14	12	100	30	5500.4	2.03E-06
43.65	14	80	20	8711.2	5.52E-06
39.44	14	80	40	8711.2	5.52E-06
40.53	14	80	60	8711.2	5.52E-06
32.12	14	90	30	7613.7	4.99E-06
33.18	14	100	40	6846.2	5.12E-06
34.64	14	110	20	6274.1	5.65E-06

Table S4. The experimental *K/S* values for nylon button at pressure, temperature, and treatment time, along with CO_2 density and solubility of dye at every pressure and temperature.

K/S	***P*/MPa**	***T*/°C**	***t*/min**	**ρ/mol・m^{-3}**	***y***
12.15	21	120	30	9632.9	4.92E-05
12.83	21	120	40	9632.9	4.92E-05
13.08	21	120	60	9632.9	4.92E-05
11.43	25	110	60	12376	9.22E-05
13.43	25	120	30	11487	9.96E-05
13.81	25	120	40	11487	9.96E-05
13.91	25	120	60	11487	9.96E-05
13.74	25	120	80	11487	9.96E-05
13.34	25	125	100	11084	1.04E-04
10.89	27	110	30	13152	1.18E-04
9.93	27	110	40	13152	1.18E-04
12.26	27	110	60	13152	1.18E-04
14.17	27	110	80	13152	1.18E-04
14.32	27	120	30	12275	1.30E-04

13.48	27	120	40	12275	1.30E-04
14.21	27	120	60	12275	1.30E-04
12.53	27	120	80	12275	1.30E-04
14.05	27	120	100	12275	1.30E-04
15.18	27	125	30	11870	1.36E-04
13.12	27	125	40	11870	1.36E-04
13.67	27	125	60	11870	1.36E-04
12.46	27	130	30	11487	1.43E-04
12.59	27	130	40	11487	1.43E-04
12.92	27	130	60	11487	1.43E-04
11.79	29	110	100	13831	1.46E-04
13.42	29	120	60	12976	1.62E-04
15.06	29	125	80	12576	1.72E-04
